# Supplementary material for: Barriers and enablers to young people accessing sexual and reproductive health services in Pacific Island Countries and Territories: A scoping review
Source: PLoS One. 2023 Jan 26;18(1):e0280667. doi: 10.1371/journal.pone.0280667 (PMC9879431; doi:10.1371/journal.pone.0280667)
Supplement: S1 Appendix — (DOCX) [file pone.0280667.s001.docx]

S1. Appendix. Scoping review protocol

**Research question**

What factors contribute to the low utilisation of sexual and reproductive health services among young people in Pacific Island Countries and Territories?

**Research aim**

To examine current literature on sexual reproductive health issues of young people in Pacific Island Countries and Territories and synthesis a narrative of barriers and enablers to sexual and reproductive health information and services.

**Title**

Barriers and enablers to young people accessing sexual and reproductive health services in Pacific Island Countries and Territories: a scoping review

**Rationale**

Young unmarried people in many Pacific Island Countries and Territories (PICTs) are not accessing sexual and reproductive health (SRH) services. Multiple factors, including cultural, religious, financial, logistical, policy, limited healthcare facilities and a shortage of healthcare workers, influence people’s access to quality SRH services [1]. In some PICTs, where SRHS are available, young unmarried people accessing those services are often stigmatised [2]. As such, young people are fearful and embarrassed about actively seeking healthcare services for contraceptives and treatment of sexually transmitted infections (STI). Equally, young unmarried people's SRH rights cannot be realised and maintained if their challenges to seek quality SRHS are not addressed.

**Objectives**

1. To examine what has been reported about the SRH of young people in PICTs
2. To examine and synthesise what has been reported on young people’s perception and practices of accessing SRH services in PICTs

**Methods**

*Design*

We will use the Preferred Reporting Items for Systematic reviews and Meta-Analyses extension for Scoping Reviews (PRISMA-ScR) checklist [3] to search three bases Medline Ovid, CINAHL Complete and Scopus. These three databases have a comprehensive overview of global literature in the fields relating to human life. The purpose is to explore young people’s accounts of their challenges in accessing SRH services in PICTs. Young people are individuals between the age ranges of 10 – 24 years, as defined by the World Health Organisation [4]. Young people in this review will include adolescents (10 -15) and youth (15-24)[5].

*Study setting*

The context for this scoping for this scoping review will be the twenty-two Pacific Island Countries and Territories (PICTs), namely; American Samoa, Cook Islands, Federated States of Micronesia, Fiji, French Polynesia, Guam, Kiribati, Marshall Islands, Nauru, New Caledonia, Niue, Northern Mariana Islands, Palau, Papua New Guinea, Pitcairn Islands, Samoa, Solomon Islands, Tokelau, Tonga, Tuvalu, Vanuatu, Wallis and Futuna. Studies conducted in Australia, New Zealand and Hawaii will not be included because these countries have higher living and socio-economic status.

*Search strategy*

Keywords and controlled vocabulary

| 1. **Sexual and reproductive health** | |
| --- | --- |
| Key words | “Sexual health” OR “reproductive health” OR contracepti* OR pregnan* OR “maternal health”OR antenatal OR postnatal OR obstetric OR delivery OR aborti* OR post-aborti* OR “family planning” OR “sexually transmitted” OR STI OR STD OR HIV OR “gender based violence” OR “intimate partner violence” |
| Controlled vocabulary | Sexual health/OR reproductive health/OR contraceptives/ OR contraception/ OR pregnancy/ OR “adolescent pregnancy”/OR maternity/OR “prenatal care”/obstetrics/OR abortion/ OR “induce abortion”/OR “family planning”/OR “sexually transmitted infection”/OR “Human immunodeficiency viruses”/OR “domestic violence” |
| **AND** | |
| 1. **Attitude to Sexual and reproductive health (knowledge, attitude and practices)** | |
| Key words | “Health knowledge” OR “Health attitude” OR “Health practice” OR “ Family planning knowledge” OR “sexual behavior” OR “Sexual partners” OR “Reproductive behavior” OR perception OR attitude* OR behavio* OR “sex education” |
| Controlled vocabulary | “ Health knowledge”/ OR “health attitude”/ OR “health practice”/ OR “Family planning knowledge”/ OR “sexual behaviour”/ OR Sexual partners”/ OR “Reproductive behaviour”/ |
| **AND** | |
| 1. **Sexual and reproductive health services** | |
| Keywords | “Health service accessibility” OR “health care” OR “Sexual health service” OR “family planning services” OR “family planning programs” OR “reproductive health service” |
| Controlled vocabulary | “Health service accessibility”/OR “health care”/ OR “Sexual health service”/ OR “family planning services”/ OR “family planning programs”/ OR “reproductive health service”/ |
| **AND** | |
| 1. **Population: Young people (10 – 24 years)** | |
| Key words | Adolescen* OR young OR youth* OR teenage* OR “young people” OR “young adult” OR student* OR “university students” OR “college students” |
| Controlled vocabulary | Adolescents/ OR adolescence/ OR teenagers/ OR “young people”/ OR “ young adults”/ OR youths/ OR “youth female”/ OR “youth male”/ OR “students” OR “college students”/OR “university students”/ OR “tertiary students”/ |
| **AND** | |
| 1. **Pacific Island Countries and Territories** | |
| Key words | “Cook Islands” OR “Federated States of Micronesia” OR Fiji OR “French Polynesia” OR “Guam” OR “Kiribati” OR “Marshall Islands” OR “ Nauru” OR “New Caledonia” OR Niue OR “Northern Mariana Islands” OR Palau OR “Papua New Guinea” OR “Pitcairn Islands” OR Samoa OR “ Solomon Islands” OR Tokelau OR Tonga OR Tuvalu OR Vanuatu OR “ Wallis and Futuna” |

*Selection of sources*

The sampling of literature will be based on the four screening stages described in PRISMA-ScR (2018) Flow Diagram. These four screening stages are;

1. **Identification**

Sources of evidences (articles) will be searched using online databases and websites. The intended databases will be Medline (Ovid), CINAHL Complete and Scopus. An additional online search will be carried out on United National Population Fund (UNFPA) and the Pacific Community websites. All published literature records from each database and website will be downloaded into Endnote (version X9) referencing software. All records will be grouped, labelled and dated. Duplicated copies of records will be removed before the start of the screening process.

**2. Screening**

Articles will be screened based on their titles, abstracts and full text. An agreement has been reached that the full text of each article will be screened by the primary investigator, followed by the other investigators. Only articles that all investigators of this scoping review have screened, and have agreed that all the elements of the inclusion criteria have been met, will be included for analysis in this scoping review.

3. **Eligibility**

All authors will screen all eligible full-text articles. Only articles that have all the elements stipulated in the inclusion criteria will be included for evaluation in this scoping

4. **Inclusion**

The remaining records of literature will be included in this study.

Identification

Records identified through database searching (n = )

Additional records identified through other sources (n = )

Records after duplicates removed (n = )

(n = )

Screening

Records screened
(n = )

Records excluded with reason (n =)

Full-text articles and grey literature assessed for eligibility (n = )

Eligibility

Full-text articles and grey literature excluded, with reasons (n= )

Included

Studies included for content analysis (n = )

*Inclusion criteria*

For records of published literature to be included in full-text screening, an article must meet the following criteria;

1. Original or primary research.

2. The records of published articles must be in English

3. The articles must have been published in the last twenty years (The year 2000 - current)

4. The article must report on

- Sexual and reproductive health issues such as contraceptive use, pregnancies, abortion, sexually transmitted infections including HIV/AIDS, comprehensive sexuality education, and sexual and reproductive health rights of young people living in PICT.
- Young people accounts of barriers and enablers of accessing sexual and reproductive healthcare services, including views and perceptions

5. The study population in published records must

- Young people (aged 10 – 24 years old) and
- Are living in one of these PICT, namely, American Samoa, Cook Islands, Federated States of Micronesia, Fiji, French Polynesia, Guam, Kiribati, Marshall Islands, Nauru, New Caledonia, Niue, Northern Mariana Islands, Palau, Papua New Guinea, Pitcairn Islands, Samoa, Solomon Islands, Tokelau, Tonga, Tuvalu, Vanuatu, Wallis and Futuna.

*Exclusion criteria*

Articles that will be excluded from full-texting screening are those that were;

1. Studies on young Pacific Island people living outside of PICT.
2. Articles on sexual and reproductive health in PICT published before 1990
3. Research conducted in PICT but not published in English
4. Conference abstracts, bulletins and newsletters about the sexual and reproductive health of young people in PICT
5. Systematic reviews
6. Young Pacific Island people living in Australia, New Zealand and Hawaii

*Main limiters*

- Date of published literature - Literature published from 2000 onwards (last twenty years covering the period of Millenium Development Goals in the Pacific through the period of Sustainable Development Goals agenda denoting sexual and reproductive health and well-being).
- Language – Only English literature

*Information sources:*

Information for this scoping review will be taken from electronic databases, namely, Scopus (multidisciplinary), Medline (Ovid), CINAHL (nursing and allied health), and from grey literature, namely, government reports, policy documents, research reports, World Health Organization Facts sheets. In addition, literature from the international organisation will be hand researched for relevant information: WHO, UNFPA, UNICEF, and UNAIDS. These international organisations have authors working on advocacy and policy documents on the sexual and reproductive health of young people. Government websites of the Australia Department of Foreign Affairs and Trade will also be explored and hand-searched for relevant information. In addition, the Australian Department of Foreign Affairs and Trade is the major Aid provider to many PICTs.

*Quality assessment*

Peer-reviewed articles and grey literatures will be appraised using these critical appraisal tools. The Joana Briggs Institutions critical appraisal checklist for qualitative research text and opinions [6]. Quantitative studies will be appraised using Critical Appraisal Skill Program (CASP) checklist [7].

*Data items*

| **Source details** | **Data** | **Comment** |
| --- | --- | --- |
| 1. Article # |  |  |
| 1. Title |  |  |
| 1. Year of publication |  |  |
| 1. Origin/country of origin (where the source was published or conducted) |  |  |
| 1. Aim/purpose |  |  |
| 1. Population and sample size |  |  |
| 1. Methodology/Method |  |  |
| 1. Intervention type, comparator and details of, e.g. duration of intervention, and duration of intervention |  |  |
| 1. Outcomes and details of how outcomes are measured |  |  |
| 1. Key findings that relate to the scoping review questions |  |  |

**Data Analysis**

The information generated in the scoping review will be analysed inductively using thematic analysis [8-10]. The purpose of inductive analysis without predetermined keywords and categories is to draw out meanings from young people’s responses. Codes will then be created and linked to form themes and a narrative of young people’s barriers and enablers to accessing SRH information and services in PICT.

**Ethical consideration**

No ethics is required for scoping reviews; however, the ethical considerations of each publication will be considered.

**Data management**

All data generated in this study will be managed according to the James Cook University data management protocol.

**Information dissemination**

I am to publish this scoping review in PLOS ONE journal.

**References**

1. Secretariat of the Pacific Community. Bridging the gap: eight perspectives on sexual and reproductive health and rights in the Pacific region. 2015. https://rrrt.spc.int/sites/default/files/resources/2019-01/SPC-SRHR_reader_ONLINE-5.pdf

2. Koster V. Sexual and reproductive rights of young people. In Secretariat of the Pacific Community. Bridging the gap: eight perspectives on sexual and reproductive health and rights in the Pacific region. 2015. https://rrrt.spc.int/sites/default/files/resources/2019-01/SPC-SRHR_reader_ONLINE-5.pdf

3.Tricco AC, Lillie E, Zarin W, O'Brien KK, Colquhoun H, Levac D, et al. PRISMA Extension for Scoping Reviews (PRISMA-ScR): Checklist and Explanation. Ann Intern Med. 2018;169(7):467-73. Epub 2018/09/05. doi: 10.7326/m18-0850. PubMed PMID: 30178033.

4.World Health Organization. Adolescent pregnancy 2020 [Internet] January 31 [cited 2021 March 31]. Available from: https://www.who.int/en/news-room/fact-sheets/detail/adolescent-pregnancy.

5.World health Organisation. Adolescent Health[Internet] .2021 [cited 2021 March 31]. Available from: https://www.who.int/southeastasia/health-topics/adolescent-health.

6. Joanna Briggs Institute. Critical appraisal tools [Internet]. 2020 [cited 2021 March 28]. https://jbi.global/critical-appraisal-tools

7. Critical Appraisal Skill Programme. CASP checklist [Internet]. 2021 [cited 2021 March 31]. Available from: https://casp-uk.net/casp-tools-checklists/

8. Braun V, Clarke V. Using thematic analysis in psychology. Qualitative Research in Psychology. 2006;3(2):77-101. doi: 10.1191/1478088706qp063oa.

9. Roller MR. A quality approach to qualitative content analysis: Similarities and differences compared to other qualitative methods. Qualitative Social Research. 2019;20(3). doi: org/10.17169/fqs-20.3.3385.

10. Hsieh H-F, Shannon SE. Three Approaches to Qualitative Content Analysis. Qualitative Health Research. 2005;15(9):1277-88. doi: 10.1177/1049732305276687.
