# Supplementary material for: Barriers and enablers to young people accessing sexual and reproductive health services in Pacific Island Countries and Territories: A scoping review
Source: PLoS One. 2023 Jan 26;18(1):e0280667. doi: 10.1371/journal.pone.0280667 (PMC9879431; doi:10.1371/journal.pone.0280667)
Supplement: S2 Appendix — (DOCX) [file pone.0280667.s002.docx]

*Appendix 2: Example of search*

The following keywords and MeSH terms were used to search the database (Medline Ovid) on the 9^th^ of September 2020.

("Sexual health" OR "reproductive health" OR contracepti* OR pregnan* OR "maternal health" OR antenatal OR postnatal OR obstetric OR delivery OR aborti* OR post-aborti* OR "family planning" OR "sexually transmitted" OR "gender based violence" OR "intimate partner violence") AND ( "Health knowledge" OR "Health attitude" OR "Health practice" OR " Family planning knowledge" OR "sexual behavior" OR "Sexual partners" OR "Reproductive behavior" OR perception OR attitude* OR  behavio* OR "sex education”) AND ("Health service accessibility" OR "health care" OR "Sexual health service" OR "family planning services"  OR  "family planning programs"  OR  "reproductive health service) AND (adolescen* OR young OR youth* OR teenage* OR "young people" OR "young adult" OR student* OR "university students" OR "college students") AND ("Cook Islands" OR "Federated States of Micronesia" OR Fiji OR  "French Polynesia" OR "Guam" OR "Kiribati" OR "Marshall Islands" OR "Nauru" OR "New Caledonia" OR niue  OR "Northern Mariana Islands" OR palau OR "Papua New Guinea" OR "Pitcairn Islands" OR samoa OR " Solomon Islands" OR tokelau OR tonga OR tuvalu OR vanuatu OR " Wallis and Futuna")

Limiters: English language, Year of publication: Year 2000 - current
