# Supplementary material for: Barriers and enablers to young people accessing sexual and reproductive health services in Pacific Island Countries and Territories: A scoping review
Source: PLoS One. 2023 Jan 26;18(1):e0280667. doi: 10.1371/journal.pone.0280667 (PMC9879431; doi:10.1371/journal.pone.0280667)
Supplement: S4 Appendix — (DOCX) [file pone.0280667.s004.docx]

S4 Appendix. 16 Items template for screening

| Date of screening: |
| --- |
| Name of screener : |
| Citation; |
| Title |
| Publication details |
| Is this a peer-reviewed article or grey literature? (Peer-reviewed or Grey literature) |
| Is the study about access to STI information and services? (Yes or No) |
| Is the study about access to HIV/AIDS information and services? (Yes or No) |
| Is the study about access to contraceptive information and services? (Yes or No) |
| Is the study about barriers and enables to accessing sexual and reproductive health services in PICTs? (Yes or No) |
| Is this study about views, beliefs, perceptions, and practices of accessing SRH information services, including healthcare-seeking behaviour? |
| Are the study participants aged between 10 – 24 years old? (Yes or No) |
| Has the study been published after 1999? (Yes or No) |
| Assessment. To be included (included), to be excluded (excluded) |
| Reasons for inclusion |
| Reasons for exclusion |
