# Supplementary material for: Barriers and enablers to young people accessing sexual and reproductive health services in Pacific Island Countries and Territories: A scoping review
Source: PLoS One. 2023 Jan 26;18(1):e0280667. doi: 10.1371/journal.pone.0280667 (PMC9879431; doi:10.1371/journal.pone.0280667)
Supplement: S5 Appendix — (DOCX) [file pone.0280667.s005.docx]

Appendix. Key Words

Barriers, Enablers, Young people, Sexual and reproductive health services, Pacific Island Countries and Territories, Scoping review.
