# Supplementary material for: Barriers and enablers to young people accessing sexual and reproductive health services in Pacific Island Countries and Territories: A scoping review
Source: PLoS One. 2023 Jan 26;18(1):e0280667. doi: 10.1371/journal.pone.0280667 (PMC9879431; doi:10.1371/journal.pone.0280667)
Supplement: Attachment — Submitted filename: Response to Reviewers.docx [file pone.0280667.s006.docx]

25^th^ of July 2022

Dear Editor,

Thank you for the invitation to respond to the reviewer's comments. Please find below a table that includes the reviewer comments, manuscript changes, and authors' notes.

If you require any further information or changes, please do not hesitate to contact me.

Kind regards,

Maggie Baigry

On behalf of the author group

**Response to reviewer comments**

| **Reviewer One** | **Changes to manuscript** | **Author notes** |
| --- | --- | --- |
| **Reviewer comments** |  |  |
| 1. First of all, it is very critical to include just eight articles for scoping review |  | Thank you for asking this important question. Eight articles met the inclusion and exclusion criteria that had been determined carefully, operationalising the aim and with a search strategy informed by a university librarian and experienced researchers. The literature review explicitly sought to examine information published in the literature that helped us to understand factors that contributed to young people's utilisation of sexual and reproductive health services in the PICTs (as per the review protocol, Appendix S1). The Pacific region has only a small population, so it is not surprising that only a small amount of literature was available to synthesise about this topic. |
| 2. Including just young people, defined as 10-20 years, seemed tricky to study about the sexual and reproductive health services. |  | Thank you for making this point. As per the literature review protocol and the methods in the manuscript, we included literature that reported on findings with populations of young people **aged 10 -24 years old. This age range is** consistent with the World Health Organization's definition of "Young people" (WHO 2021) and with the social and cultural context of the Pacific island country and territory settings being included in the study.  Reference: World Health Organization. Adolescent pregnancy 2020 [Internet] January 31 [cited 2021 March 31]. Available from: https://www.who.int/en/news-room/fact-sheets/detail/adolescent-pregnancy. |
| 3. The study has talked about the actions of health care providers giving condoms and contraceptives to only married men and women, affirming the perception that only married people were eligible to access family health services (result: lack of sexual and reproductive health knowledge). However, the study included the participants aged 10-20 years. Should young people aged, for example, aged 10-15~16 years aged be given the contraceptives from the health care providers? Is the policy of PICTs support this? |  | Thank you for your reflection on our findings from the review. These findings are evidenced in the literature and consistent with the social and cultural context in which the studies took place. The results demonstrate the exclusion of young people who are not married and provide evidence of supporting changes to policy and practice for equitable health service delivery.  (Dawson et al., 2021) for example, reported that there are policies that support young, unmarried people accessing contraceptives, in reality, the social and cultural barriers to delivering such services mean young people are predominantly excluded from accessing contraceptives (Raman et al., 2015). |
| - Also, the age group identified in this study mostly comprises of the unmarried population (or say too early to get married), so it is unlikely to compare these results with the married population. |  | Thank you for this important point. The scoping review methodology (Tricco et al., 2018) did not formally seek to compare married and unmarried populations but to describe the published literature findings that met the selection criteria. However, as reported on page 10, for example, it became evident from the review data that unmarried people were mostly unable to access contraceptives. |
| 4. The themes created to portray Barriers and Enabler of SRH in PICTs shows the strength of the study; however, the articles itself contradict the information provided in the articles; - For example; the behaviour of health care providers is shown as both barrier and an enabler - Similarly, the parent knowledge and perception were also shown as both barriers and enablers |  | Thank you for this comment. You have highlighted the social and cultural complexities of SRH for young people of the Pacific. We have sought to show that multi-factorial drivers are impacting the access to SRH services - for some young people, their parents were supportive and enabled them to access SRH services (p.14), while other parents did not provide relevant SRH support, and this was a barrier. We have sought to report all evidence provided in the included literature and show the complexity of situations young people encounter. |
| 5. It is recommended to include the late adolescents (rather than 10-20 years) in the study, which would actually show the barriers and enablers of SRH, since it is too early for the early adolescent to seek/access and utilise SRH (by themselves) |  | We understand from the WHO definition (WHO 2021) that the age group of 10-24 years, as included on p. xx, includes late adolescents. While the inclusion of 10-24 years was a wide range in terms of the development of a young person, the predominant age of populations reported in articles in the review were 15 years and above, the age group that would be most likely to access SRH. |
| 6. Referencing should be checked since some of the references is missing (ref. 37 and directly ref. 55 is shown > ref 38-54 is missing) | page 18 - 19 | Thank you for pointing this out. Referencing is very important, and we have worked on and updated our in-text citation and reference list. |
| 7. I am not fully authorised for the English language checking but, some grammatical corrections is recommended in some sentences |  | Thank you for this response. We have carefully reviewed the manuscript and made some small changes to ensure excellent grammar throughout. |

Dawson, A., Ekeroma, A., Wilson, D., Noovao-Hill, A., Panisi, L., Takala, B., Black, K., & Bateson, D. (2021). How do Pacific Island countries add up on contraception, abortion and reproductive coercion? Guidance from the Guttmacher report on investing in sexual and reproductive health. *Reproductive Health*, *18*(1), 68. <https://doi.org/10.1186/s12978-021-01122-x>

Raman, S., Nicholls, R., Pitakaka, F., Gapirongo, K., & Hou, L. (2015). Identifying and overcoming barriers to adolescent sexual and reproductive health: perspectives and practices of health, education and welfare workers. *Pacific Journal of Reproductive Health*, *1*, 4-13. <https://doi.org/10.18313/pjrh.2015.1000>
