# Supplementary material for: Barriers and enablers to young people accessing sexual and reproductive health services in Pacific Island Countries and Territories: A scoping review
Source: PLoS One. 2023 Jan 26;18(1):e0280667. doi: 10.1371/journal.pone.0280667 (PMC9879431; doi:10.1371/journal.pone.0280667)
Supplement: Attachment — Submitted filename: Response to reviewer 11.11.22.docx [file pone.0280667.s007.docx]

11^th^ November 2022

Dear Editor,

Thank you for the invitation to respond to the reviewer's comments. Please find below a table that includes the reviewer's comments, manuscript changes, and authors' notes.

If you require any further information or changes, please do not hesitate to contact me.

Kind regards,

Maggie Baigry

On behalf of the author group

Response to the second reviewer's comments

| **Reviewer two** | **Changes to manuscript** | **Author notes** |
| --- | --- | --- |
| **Reviewer comments** |  |  |
| 1) Please state in the text what other sources were searched to identify the 40 papers included in the Identification section of Figure 1. This would be best inserted after the sentence about the databases searched. | Added another sentence to the first paragraph on page 5 | Thank you for raising this important point and pointing us to where we need to specify more information in the text.  The other sources of information were google scholar, PubMed, Web of Science, and JCU OneSearch. Since this scoping review focuses primarily on young people's responses (stories, experiences), we eliminated second (parents, teachers) and third-party (government reports) responses. |
| 2) Please check that the two papers attributed to Kennedy are separate studies and are not reporting the same data from the same study (and, therefore, the same study population). Likewise, for the two papers by White, if so, you will need to address this in the limitations as it could skew the conclusion you draw from the data.  This is a particularly important methodological issue for themes where the evidence is only derived from one study population rather than reported across multiple study populations (e.g. the theme "Friendly non-judgemental and kind healthcare providers" is only supported by data published by Kennedy). | We added a sentence to the first paragraph on page 7 | Thank you for asking this important question about the number of articles included in this scoping review. To answer the question,  Of the eight articles, two by Kennedy et al. (2013, 2014) reported data from one population. Likewise, two studies by White (2018a, 2018b) were also based on one population.”  We acknowledged that we did not clearly state it in our results and have now made the clarification in the text. |
| 3) Can the authors clarify in the manuscript whether they used original quotes from the papers or the themes derived and reported in the papers as the data for the thematic analysis? | On page 6, we added a sentence to clarify our data screening and charting process. | Thank you for asking us to clarify this important point about the use of original quotes and themes in our findings.  First, we did not use original quotes in our manuscript. Instead, we used themes and quotes reported in the eight articles as our data source for the inductive thematic analysis. We try as much to put forward the voices of the young people in each of the eight articles.  Second, the themes in our text were derived from the inductive analysis of the eight papers. |
| 4) Can the authors include the number of reviewers for each step of the data screening process and include their initials in brackets in the text?  Also, can the authors include who did the thematic analysis and include their initials in brackets in the text? | Initials of authors were added to the text. Page 7 | Thank you for asking us this question. Our initials have been added to the text. |
